# Exploring the prospects, advancements, and challenges of *in vitro* modeling of the heart-brain axis

**DOI:** 10.3389/fncel.2024.1386355

**Published:** 2024-05-03

**Authors:** Senegal Alfred Mabry, Narciso Pavon

**Affiliations:** ^1^Affect and Cognition Laboratory, Department of Psychology and Human Development, College of Human Ecology, Cornell University, Ithaca, NY, United States; ^2^ChangHui Pak Laboratory, Department of Biochemistry and Molecular Biology, College of Natural Sciences, University of Massachusetts-Amherst, Amherst, MA, United States

**Keywords:** assembloid, organoid, heart, brain, autonomic nervous system, induced pluripotent stem cells, sympathetic, cardiomyocyte

## Abstract

Research on bidirectional communication between the heart and brain has often relied on studies involving nonhuman animals. Dependance on animal models offer limited applicability to humans and a lack of high-throughput screening. Recently, the field of 3D cell biology, specifically organoid technology, has rapidly emerged as a valuable tool for studying interactions across organ systems, i.e., gut-brain axis. The initial success of organoid models indicates the usefulness of 3D cultures for elucidating the intricate interactivity of the autonomic nervous system and overall health. This perspective aims to explore the potential of advancing *in vitro* modeling of the heart-brain axis by discussing the benefits, applications, and adaptability of organoid technologies. We closely examine the current state of brain organoids in conjunction with the advancements of cardiac organoids. Moreover, we explore the use of combined organoid systems to investigate pathophysiology and provide a platform for treatment discovery. Finally, we address the challenges that accompany the use of 3D models for studying the heart-brain axis with an emphasis on generating tailored engineering strategies for further refinement of dynamic organ system modeling *in vitro*.

## 1 Introduction

The heart and the brain have a mutually indispensable relationship. Dysfunction in one organ system causing pathological changes in the other are clinical implications of the heart-brain axis (Manea et al., 2015; Tahsili-Fahadan and Geocadin, 2017; Sha et al., 2023), but their interconnectedness is deeper. Brain and heart development occurs early and almost simultaneously in organogenesis (Donovan and Cascella, 2024). The precise dance of timing and signaling in neurogenesis and cardiogenesis is regulated by genes for progenitor proliferation (Sonic Hedgehog) and cellular commitment (notch, jagged, Nkx2.5), shared among various organs, but that have exclusive roles in shaping heart and brain development (Simeone et al., 1995; Dessaud et al., 2008). Nearly half of congenital heart issues are linked to complications in genetics (Wnt, Sonic Hedgehog, Bone Morphogenetic Protein) that aid neural crest development, heart septation, and cell migration (Dessaud et al., 2007, 2008; Yu et al., 2008; Buijtendijk et al., 2020).

A network of brain regions, including the hypothalamus, cerebellum, amygdala, and insula, make up the central autonomic network. Sympathetic, parasympathetic, and sensory nerves abundantly innervate the heart (Fukuda et al., 2015), and their direct influence over the sinoatrial and atrioventricular nodes supports the contraction of heart muscles and the pumping of blood. The brain regions in the central autonomic network regulate sympathetic and parasympathetic heart activity through afferent and efferent vagal pathways (Silvani et al., 2016). Vagal efferents modulate cardiac activity, but the nerve is primarily composed of afferents (70%), which produce Calcitonin gene-related peptides in the heart that can cause adverse cardiac remodeling or have cardioprotective effects (Berthoud and Neuhuber, 2000; Chai et al., 2006; Meléndez et al., 2011; Habecker et al., 2016). The cerebellum and the hypothalamus receive cardiac signals from heart sensory nerves. They can both modulate heart rate or ejection fraction, for example, through the critical Hypothalamic–Pituitary–Adrenal Axis, or they can relay cardiac signals elsewhere in the brain (Sheng et al., 2021; Neubert et al., 2023). The amygdala and insula receive cardiac signals, interpret autonomic arousal, and then modulate sympathetic and parasympathetic activity to meet emotional needs (Chouchou et al., 2019). The locus coeruleus in the hindbrain, which produces norepinephrine for the brain, can also increase sympathetic activity or decrease parasympathetic activity in cardiac nerves and is implicated in genetic and stress-induced cardiovascular diseases (Wood and Valentino, 2017; Lian et al., 2023).

The peculiar and powerful ways that brain or heart affliction disrupts the other system indicate that their partnership is unique. We recognize that the body is an integrated system, and it follows that if there is an injury or developmental abnormality in one organ, others may be affected. But why and how, in Takotsubo syndrome, neural activity like extreme psychological stress or the hyperconnectivity of autonomic brain regions may cause the fully formed left ventricle to balloon to a different shape in adulthood goes unexplained with our current knowledge of organ crosstalk (Rossi et al., 2022). Other puzzling cases of heart-brain axis dysfunction are numerous and occur across the lifespan. Parkinson’s disease patients have been subtyped into body-first or brain-first disease trajectories using multimodal images of the heart and brain (Horsager et al., 2020), but it is unclear why and how the heart would be a propagation site for α-synuclein (Van Den Berge and Ulusoy, 2022). High AB amyloid levels in familial hypercholesterolemia patients and risk for mild cognitive impairment exceed age predictors and sporadic hypertension control groups (Zambón et al., 2010). Genetic and *in-utero* environmental complications cause congenital heart diseases with severe neurodevelopmental issues (Sha et al., 2023) and lifelong impacts on heart and brain development.

**Figure 1:**
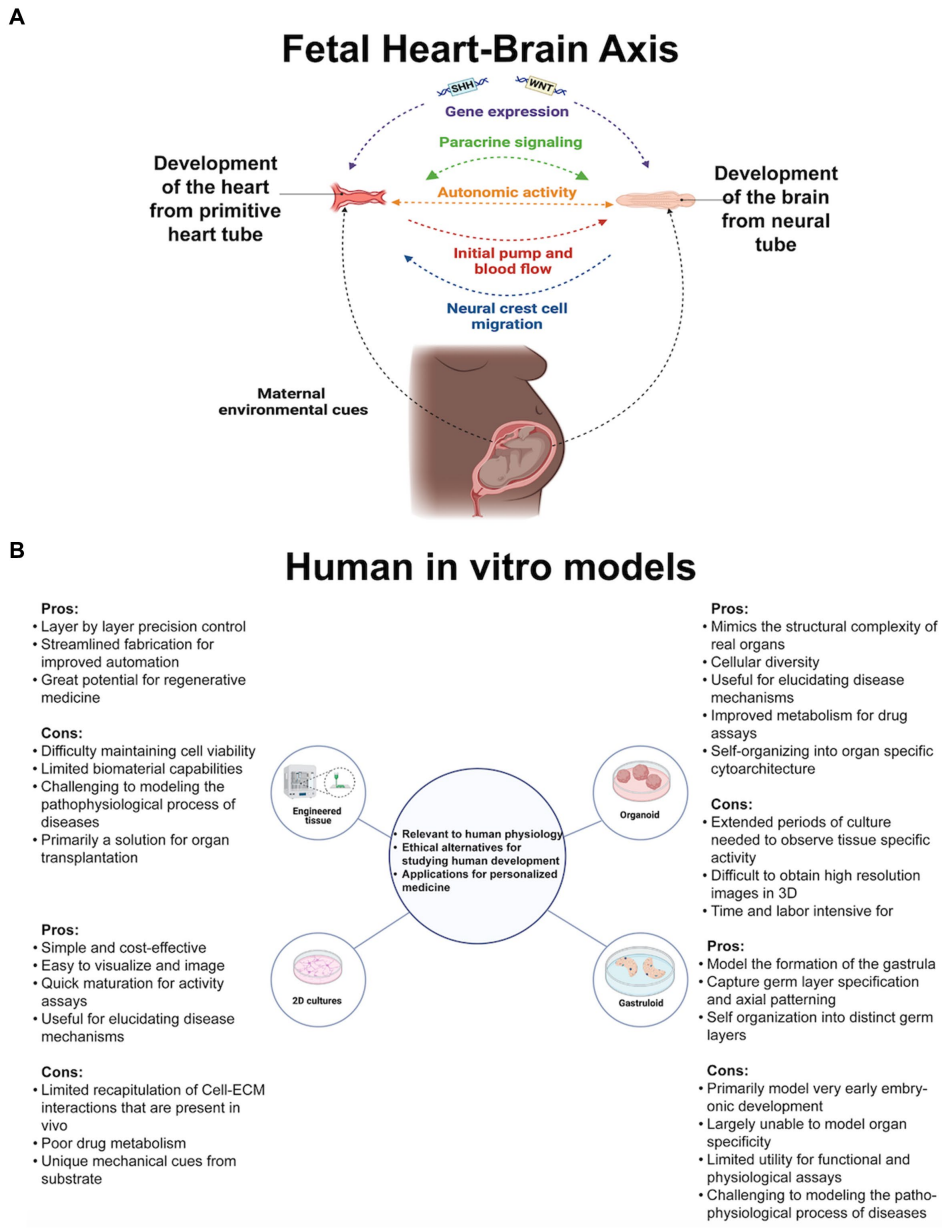
**(A)** Genetic and environmental cues guide cell proliferation of the earliest heart-brain-like structures. Neural crest cell migration innervates the heart with sympathetic, parasympathetic, and sensory nerves. The heart and the placenta support the brain’s growth cycle with oxygen autonomically because their mutual relationship has been formed through cell connections and paracrine signaling. Genetic, structural, autonomic, and cell communication factors are essential elements of heart-brain communication postnatally. **(B)** Researchers’ questions primarily determine their cell culturing approach. Organoids, the self-assembling 3D model that can be connected with other tissue to form assembloids, may be the most useful for investigating heart-brain connections because of their developmental and functional capabilities. Figure created using Biorender (https://biorender.com/).

Identifying how the partnership between the heart and brain influences development, (see Figure 1A), and disease is difficult for researchers to do *in vivo*. Processes causing heart and brain interdependence are challenging to identify outside of fetal development because as neonates’ human hearts and brains shift from cell proliferation to maturation. In humans, protein expression differs from mice (Lin et al., 2014), and there are tremendous temporal and genetic expression differences in heart-brain disorders (Pervolaraki et al., 2013, 2018). Failure in fetal heart adaptations that support brain oxygenation, e.g., placenta formation, disrupts the brain growth cycle early in development (Miller et al., 2016; Zhang and Lindsey, 2023). The changes are so early that neuroimaging checking for abnormalities comes too late in cases of congenital heart disease or from *in-utero* environmental factors like gestational diabetes, prenatal stress, and preeclampsia (Limperopoulos et al., 2010; McQuillen and Miller, 2010). We believe a functional fetal model of the human heart-brain axis can be built *in vitro* to uncover meaningful hidden relationships across the lifespan.

## 2 *In vitro* organ modeling capabilities and their heart-brain axis applications

### 2.1 Current state of *in vitro* modeling for organ connections and dysfunction

Organ cells cultured in laboratories recapitulate fetal tissue properties and functions. Cultures are embryogenic copies of organs generated from stem cells (embryonic or pluripotent) donated by healthy humans or clinical patients or taken from animals for genetic and tissue property testing. See Figure 1B for the types of cultures referenced throughout the perspective. For *in vitro* culturing and analysis fundamentals, see Duval et al. (2017), then return here for heart and brain modeling specifics. Before delving into heart-brain implications, it is crucial to highlight the advanced capabilities of *in vitro* models. Researchers have started using two or more connected organoids, known as assembloids, for modeling interactions between multiple organ systems. Assembloids have recapitulated cortico-thalamic, cortical-subpallium, cortico-striatal, gut-brain, visual system, and hindbrain-skeletal muscle interactions (Bagley et al., 2017; Birey et al., 2017; Xiang et al., 2019; Andersen et al., 2020; Miura et al., 2020; Fligor et al., 2021; Reiner et al., 2021). Morphogens guide cell fate differentiation, enhance reproducibility, and yield brain organoids with specific cell fates (Muguruma et al., 2015; Birey et al., 2017; Xiang et al., 2017, 2019) and cardiac organoids with neurons or endothelial cells resembling fetal heart development (Marini et al., 2022). Culturing adult cell types is difficult, but *in vitro* modeling of Huntington’s and Parkinson’s has identified early markers of these midlife or late-life diseases (Smits et al., 2019; Andrews and Kriegstein, 2022).

An exemplar of *in vitro* model effectiveness comes from research on tuberous sclerosis complex. This disorder afflicts adults and children with numerous benign tumors in different organ systems, and brain tumors can lead to severe neurological issues (Henske et al., 2016). Animals cannot appropriately model pathognomonic lesions in the disease, and postmortem patient tissue rarely reveals loss of heterozygosity, an important biomarker, in dysplastic tissues. So, Eichmüller et al. (2022) cultured brain organoids from patients with tuberous sclerosis complex genetic mutations and, in recreating fetal processes, captured the emergence of brain tumors and identified the progenitor population that gives rise to them. Moreover, an integrated model of the heart and brain would be particularly beneficial for this research, as tumors associated with tuberous sclerosis complex often manifest first in the heart (Hinton et al., 2014). However, unlike in the brain, they fade (Will et al., 2023) and rarely lead to severe issues. Replaying fetal development in integrated models may unravel organs’ developmental journeys to reveal insights into development and disease.

### 2.2 *In vitro* models of heart-brain axis

We limit this perspective to findings from using or building *in vitro* tools to uncover more about the exclusive relationship between the heart and the brain. Firstly, we share insights from modeling sympathetic neuron control of cardiomyocytes in traditional cell cultures. The sympathetic-cardiomyocyte connection is a direct pathway for heart-brain axis communication, but there are limitations inherent in using traditional cultures for this modeling. Secondly, we explore 3D models for findings that cannot be achieved in traditional cultures but which stop short of accurately modeling development and integrated function of the human heart and brain. Lastly, we discuss the feasibility of creating human heart-brain assembloids (hHBAs) to develop the model most comparable to organ development and function, as the human 3D tool has yet to be introduced.

## 3 Limitations and insights from modeling sympathetic cardiomyocyte regulation in traditional cell cultures

### 3.1 Traditional cultures limit faithful modeling of sympathetic-cardiomyocyte tissue features

In the heart-brain axis, connections between sympathetic and parasympathetic neurons and cardiomyocytes provide direct pathways for neural regulation of cardiac functions such as contractility and automaticity (Gordan et al., 2015). Cultures can recapitulate sympathetic efferent control of the heart. Oh et al. (2016) first stimulated mouse sympathetic neurons with nicotine and measured increased beating in co-cultured myocytes. Modeling efferent control of the heart is needed to investigate the development of the partnership between the heart and the brain. Autonomic breakdowns can occur as early as embryogenesis (Al Nafisi et al., 2013; Siddiqui et al., 2015), the malformation of sympathetic neurons is linked to prenatal heart failure and postnatal arrhythmia (Tada et al., 2007; Brack et al., 2013). On the one hand, research into sympathetic control of cardiomyocytes in traditional cultures has yielded findings into how heart and brain development is codependent and relevant for disease. On the other hand, this research in traditional cultures faces limitations, especially when compared to more advanced 3D models that have addressed issues such as no cell-environment interactions (Cukierman et al., 2001; Ravi et al., 2015), decreased cell junctions (Soares et al., 2012), and unnaturally rapid cell proliferation with poor differentiation (Ravi et al., 2015; Costa et al., 2016; Langhans, 2018).

We explain the shortcomings of traditional cultures in sympathetic neuron cardiomyocyte modeling first. Traditional cultures cannot recapitulate details of heart-brain interactions because they lack cell-matrix interactions, preventing them from forming complex tissue structures. Research in traditional cultures like Takayama et al. (2020), where nicotine-stimulated parasympathetic and sympathetic neurons inversely alter cardiomyocyte beating, is less faithful to *in vivo* processes because organ structure cannot be replicated. In parasympathetic regulation of the heart, the neurons are located in the epicardium and regulate activity in the sinoatrial and atrioventricular nodes, which have functions and tissue properties specific to heartbeat regulation that are not accounted for in traditional cultures (Stavrakis and Po, 2017). In 3D cultures, guided differentiation with cell-matrix interactions engenders fetal-like heart structures (left ventricle, right ventricle, atria, outflow tract, and the atrioventricular canal), providing a tool to test function and structure (Schmidt et al., 2023). Additionally, different gradients of the sympathetic innervation of heart regions cannot be modeled in traditional cultures (Volmert et al., 2023). Sympathetic innervation gradients in cardiac nodes are linked to mouse bradycardia and likely influence brain-heart control (Chow et al., 1993; Kawano et al., 2003; Maden et al., 2012; Tillo et al., 2012; Hasan, 2013).

### 3.2 Findings from traditional cultures could be reproduced with 3D models

Modeling sympathetic regulation of cardiomyocytes in traditional cultures underscores the significance of sympathetic and parasympathetic neuron plasticity in development and disease (Kanazawa and Fukuda, 2022). Traditional cultures enhance researchers’ capacity to model features of organ interconnections. However, 3D culture research could replicate these findings while addressing structural limitations inherent in traditional cultures. In experiments with sympathetic mouse neuron cultures, Larsen et al. (2016) found that the condition of the neurons (healthy or pro-hypertensive) affected heart cell responses, not the condition of the co-cultured human heart cells. Their experiments found that genes crucial for heart development were more active in mouse neurons and human cardiomyocyte co-culture than in cardiomyocytes alone. Norepinephrine is one of the main neurochemicals exerting control over the heart (Elia and Fossati, 2023). Winbo et al. (2020) add neuromodulatory capabilities to sympathetic-cardiomyocyte modeling by co-culturing norepinephrine-secreting sympathetic neurons with cardiomyocytes and demonstrating that cardiomyocytes increased beating after neuronal nicotine stimulation. Nevertheless, single and multi-organ 3D cultures of the heart and brain have led to research findings that traditional cultures could not have uncovered.

## 4 Advantages, insights, and considerations in modeling the human heart-brain axis in 3D cultures up to the early fetal period

### 4.1 Advantages of 3D cultures, particularly organoids for reproducing development features and fetal organ function

Culturing 3D tissues, especially organoids, yields insights into heart and brain interdependence that are not achievable in other systems because they provide a more accurate representation of development and human physiological responses (Haisler et al., 2013; Imamura et al., 2015; Voges et al., 2017; Menasché et al., 2018). Advancements in cardiac organoids enable the discovery of specific organ interconnections and functional features. Hofbauer et al. (2021) cultured cardiac organoids that developed endocardial cells and a chamber-like cavity, a feature other tools, particularly traditional cultures, cannot replicate. Lewis-Israeli et al. (2021) cultured cardiac organoids with epicardium, endocardium, and cardiac fibroblast cell types. The ability to culture cardiac organoids with heart chamber-like cavities and diverse cell types is crucial for faithfully modeling how the heart and brain become intertwined in development. Through mechanobiological processes, cross-talk between the brain, early heart structures, epicardium, and endocardial cells influences cytodifferentiation and organ pathophysiology. With organoids, developmental plasticity is often coupled with appropriate organ physiological responses. Hofbauer et al. (2021) leveraged that benefit to demonstrate fetal cardiac regeneration capabilities, and Lewis-Israeli et al. (2021) demonstrated how *in-utero* environmental differences cause cardiac structure abnormalities.

### 4.2 Insights and considerations in relevant 3D multi-organ modeling of heart and brain interconnections

**Figure 2:**
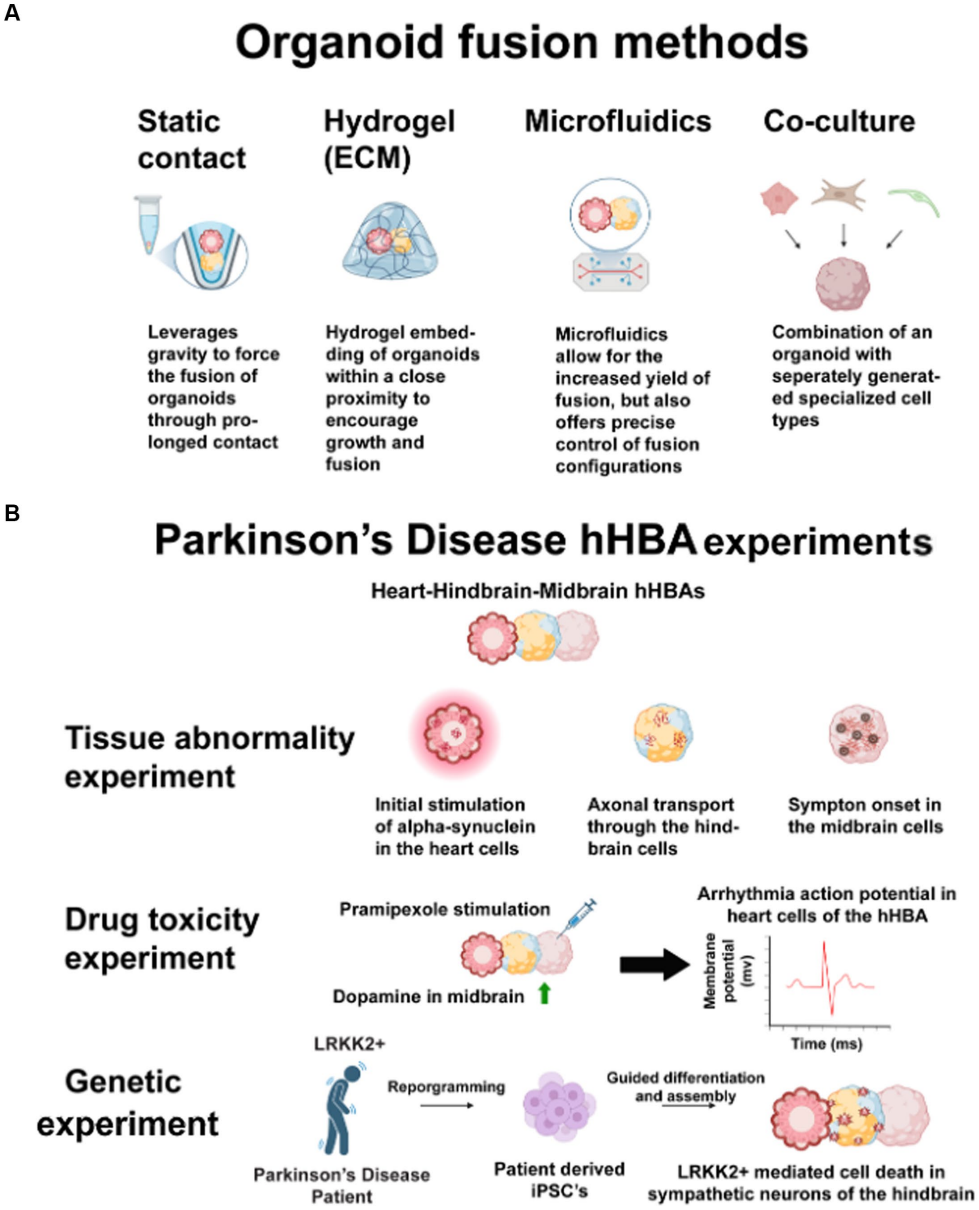
**(A)** These techniques have the same benefits for connecting heart-brain organoids into assembloids. Researchers may use static contact or microfluidics to avoid potential issues with different ECMs across multiple organoids. Co-culturing may negate the structural benefits of 3D cultures. **(B)** Parkinson’s disease has many opaque heart-brain connections. Heart, hindbrain, and midbrain cells connected in one hHBA could answer these three questions about the disease. First, introducing human alpha-synuclein to heart cells and observing propagation in a hHBA could show how alpha-synuclein spreads through the sympathetic nervous system. Second, stimulating midbrain cells with Pramipexole may show how some pharmaceutical interventions increase dopamine in midbrain cells but cause cardiotoxicity. Third, by culturing LRRK2+, patient-derived-iPSCs may show genetic connections in midbrain cell death and sympathetic denervation. Figure created using Biorender (https://biorender.com/).

The fidelity of 3D cultures to fetal organ development processes and function sparked a new goal among researchers: to culture 3D multi-organ models (Picollet-D’hahan et al., 2021). Researchers interested in modeling the human gut-brain axis *in vitro* have successfully used assembloids to uncover meaningful hidden connections between systems (Bellono et al., 2017; Kim et al., 2021; Trapecar et al., 2021). See Figure 2A for the different techniques for connecting organoids. Bellono et al. (2017) demonstrated how enterochromaffin chemoreceptors communicate environmental and homeostatic processes from the gut directly to the nervous system, and Trapecar et al. (2021) discovered gene expression mechanisms for neuron, astrocyte, and microglia maturation and function. Despite these successes in uncovering links between organ systems using assembloids for gut-brain dynamics, a human heart-brain assembloid (hHBA) has yet to be cultured and tested. Instead, the most relevant human multi-organ model of the heart and brain was built using gastruloids that mimic early embryogenic, not fetal, organ development and lack functional capabilities (Olmsted and Paluh, 2022). While there is room for critique on how models were built or tested, many 3D multi-organ models have findings relevant to uncovering heart-brain dynamics or culturing a future hHBA.

In chronological order, Skardal et al. (2020) co-cultured assembloid of the liver, lung, testes, heart, and brain and revealed cardiotoxicity in cells exposed to drugs recalled by the US Food and Drug Administration. Their work demonstrates the feasibility of integrating models of the heart and brain, but the authors do not test neuro-cardiac interconnectedness in their model. Then, Rossi et al. (2021) cultured mouse gastruloids and discovered that regulatory factors from non-cardiomyocytes, such as vascular endothelial growth factors and fibroblast growth factors, could influence cardiomyocyte beating. Without providing specific heart-brain insights, this work is still relevant for future neuro-cardiac modeling because it introduced a new engineering approach and demonstrated how other cell factors influence cardiomyocytes (Yang et al., 2020). Later, Drakhlis et al. (2021) and Silva et al. (2021) cultured heart-gut multi-organ models and, respectively, showed foregut cells interweaved and scattered throughout the cardiac tissue and a transcriptional state roughly equidistant from cardiac and gut progenitors. Even without direct heart and brain implications, these models illustrate the tissue and genetic differences in 3D multi-organ models. Later, Olmsted and Paluh (2022) showed gastruloids with neuro-cardiac cooperation precursors, e.g., neuropeptide Y and brain-derived neurotrophic factor, exhibiting a more autonomic transcriptomic phenotype compared to gastruloids lacking cardiac signaling. However, gastruloids are early embryonic models unsuitable for testing functional organ coupling. Liu et al. (2023) co-cultured reprogrammed mouse fibroblasts, transformed into autonomic ganglion organoids with neonatal mouse ventricular myocytes. This research is just short of an hHBA, and excitingly, stimulation of the autonomic ganglions with nicotine increased mouse myocyte beating, replicating the findings from traditional cultures. Lastly, Hernandez et al. (2023) bioprinted neuronal Parkinson’s model cells and cardiomyocytes to create a tissue development platform. However, this may be more useful in the future as an intervention than for uncoupling brain and heart connections.

## 5 Discussion: considerations in culturing hHBAs and culturing hHBAs from patient-iPSCs with heart and brain abnormalities

This perspective is timely to offer guidance for future human heart-brain *in vitro* research because a tool for investigating organ connections during development *and* disease has yet to be introduced. Humans have different developmental timelines and transcriptomics than mice (Anzai et al., 2020), and human tissue may prove more beneficial for drug testing than animals (Varga et al., 2015; Van Norman, 2019). Addressing media needs for different types of connected tissues may be challenging, but issues will likely be specific to researchers’ design choices. Design standards have been difficult to set for *in vitro* models because of researchers’ diverse questions and the almost unlimited flexibility in what can be cultured. Uncoupling the role of the autonomic nervous system in the heart-brain axis may be of keen interest to researchers who culture hHBAs. However, there are also applications for genetic, tissue abnormality, and drug toxicity experiments. Figure 2B illustrates the design of an hHBA cultured to study all those areas in Parkinson’s disease. Clear rationale is crucial to ensure dissimilar hHBA models still better the field’s understanding of organ interconnectedness (Cho et al., 2022). The likely use of patient cells to study the heart-brain axis heightens the case for clear design choices. While patient-iPSCs may influence developmental signaling pathways unexpectedly, they may also accelerate research.

Brain diseases can affect the heart via genetics and autonomic processes, and vice versa for heart diseases. With patient-iPSCs, pathological dysfunctions in heart and brain tissues could be used to identify any interrelationship between abnormalities using hHBAs. For example, SCN1A gene expression is associated with sudden unexplained death in epilepsy (SUDEP), which has unclear connections to the heart and the brain. Patients with Dravet syndrome, a rare genetic encephalopathy, have a higher risk for SUDEP (Kearney, 2013) and often have variations in the SCN1A gene. Dravet syndrome patient-iPSCs develop cardiac (Frasier et al., 2018) and neural (Higurashi et al., 2013) abnormalities in ways indicating neuronal hyperexcitability and predisposition for arrhythmia are caused by SCN1A haploinsufficiency. Culturing an hHBA from Dravet syndrome patient-iPSCs will more closely resemble *in vivo* developmental changes in structure and function from SCN1A expression. SCN1A is expressed in both the heart and brain (Malhotra et al., 2001; Mishra et al., 2015), so only an hHBA can elucidate when, where, and how SCN1A gene expression starts to affect and intertwine systems pathologically. Culturing an hHBA from patient cells allows researchers to closely mimic human organ processes in a connected system. If researchers are going to enhance the field’s understanding of the heart-brain axis, then research on *in vitro* systems that can tackle the complexity of their partnership must advance.

## Author contributions

SAM: Conceptualization, Project administration, Writing – original draft, Writing – review & editing. NP: Visualization, Writing – original draft, Writing – review & editing.

## References

[ref1] Al Nafisi B. Van Amerom J. F. Forsey J. Jaeggi E. Grosse-Wortmann L. Yoo S.-J.. (2013). Fetal circulation in left-sided congenital heart disease measured by cardiovascular magnetic resonance: a case–control study. J. Cardiovasc. Magn. Reson. 15:65. doi: 10.1186/1532-429X-15-65, PMID: 23890187 PMC3735489

[ref2] Andersen J. Revah O. Miura Y. Thom N. Amin N. D. Kelley K. W.. (2020). Generation of functional human 3D Cortico-motor Assembloids. Cell 183, 1913–1929.e26. doi: 10.1016/j.cell.2020.11.017, PMID: 33333020 PMC8711252

[ref3] Andrews M. G. Kriegstein A. R. (2022). Challenges of organoid research. Annu. Rev. Neurosci. 45, 23–39. doi: 10.1146/annurev-neuro-111020-090812, PMID: 34985918 PMC10559943

[ref4] Anzai T. Yamagata T. Uosaki H. (2020). Comparative transcriptome landscape of mouse and human hearts. Front. Cell and Develop. Biol. 8:268. doi: 10.3389/fcell.2020.00268, PMID: 32391358 PMC7188931

[ref5] Bagley J. A. Reumann D. Bian S. Lévi-Strauss J. Knoblich J. A. (2017). Fused cerebral organoids model interactions between brain regions. Nat. Methods 14, 743–751. doi: 10.1038/nmeth.4304, PMID: 28504681 PMC5540177

[ref6] Bellono N. W. Bayrer J. R. Leitch D. B. Castro J. Zhang C. O’Donnell T. A.. (2017). Enterochromaffin cells are gut Chemosensors that couple to sensory neural pathways. Cell 170, 185–198.e16. doi: 10.1016/j.cell.2017.05.034, PMID: 28648659 PMC5839326

[ref7] Berthoud H.-R. Neuhuber W. L. (2000). Functional and chemical anatomy of the afferent vagal system. Auton. Neurosci. 85, 1–17. doi: 10.1016/S1566-0702(00)00215-0 11189015

[ref8] Birey F. Andersen J. Makinson C. D. Islam S. Wei W. Huber N.. (2017). Assembly of functionally integrated human forebrain spheroids. Nature 545, 54–59. doi: 10.1038/nature22330, PMID: 28445465 PMC5805137

[ref9] Brack K. E. Winter J. Ng G. A. (2013). Mechanisms underlying the autonomic modulation of ventricular fibrillation initiation—tentative prophylactic properties of vagus nerve stimulation on malignant arrhythmias in heart failure. Heart Fail. Rev. 18, 389–408. doi: 10.1007/s10741-012-9314-2, PMID: 22678767 PMC3677978

[ref10] Buijtendijk M. F. J. Barnett P. Van Den Hoff M. J. B. (2020). Development of the human heart. Am. J. Med. Genet. C: Semin. Med. Genet. 184, 7–22. doi: 10.1002/ajmg.c.31778, PMID: 32048790 PMC7078965

[ref11] Chai W. Mehrotra S. Jan Danser A. H. Schoemaker R. G. (2006). The role of calcitonin gene-related peptide (CGRP) in ischemic preconditioning in isolated rat hearts. Eur. J. Pharmacol. 531, 246–253. doi: 10.1016/j.ejphar.2005.12.039, PMID: 16438955

[ref13] Cho S. Discher D. E. Leong K. W. Vunjak-Novakovic G. Wu J. C. (2022). Challenges and opportunities for the next generation of cardiovascular tissue engineering. Nat. Methods 19, 1064–1071. doi: 10.1038/s41592-022-01591-3 36064773 PMC12061062

[ref14] Chouchou F. Mauguière F. Vallayer O. Catenoix H. Isnard J. Montavont A.. (2019). How the insula speaks to the heart: cardiac responses to insular stimulation in humans. Hum. Brain Mapp. 40, 2611–2622. doi: 10.1002/hbm.24548, PMID: 30815964 PMC6865697

[ref15] Chow L. T. Chow S. S. Anderson R. H. Gosling J. A. (1993). Innervation of the human cardiac conduction system at birth. Heart 69, 430–435. doi: 10.1136/hrt.69.5.430, PMID: 7686024 PMC1025107

[ref16] Costa E. C. Moreira A. F. De Melo-Diogo D. Gaspar V. M. Carvalho M. P. Correia I. J. (2016). 3D tumor spheroids: An overview on the tools and techniques used for their analysis. Biotechnol. Adv. 34, 1427–1441. doi: 10.1016/j.biotechadv.2016.11.002, PMID: 27845258

[ref18] Cukierman E. Pankov R. Stevens D. R. Yamada K. M. (2001). Taking cell-matrix adhesions to the third dimension. Science 294, 1708–1712. doi: 10.1126/science.1064829, PMID: 11721053

[ref19] Dessaud E. McMahon A. P. Briscoe J. (2008). Pattern formation in the vertebrate neural tube: a sonic hedgehog morphogen-regulated transcriptional network. Development 135, 2489–2503. doi: 10.1242/dev.009324, PMID: 18621990

[ref20] Dessaud E. Yang L. L. Hill K. Cox B. Ulloa F. Ribeiro A.. (2007). Interpretation of the sonic hedgehog morphogen gradient by a temporal adaptation mechanism. Nature 450, 717–720. doi: 10.1038/nature06347 18046410

[ref22] Donovan M. F. Cascella M. (2024). Embryology, weeks 6–8. In StatPearls. StatPearls Publishing. Available at: http://www.ncbi.nlm.nih.gov/books/NBK563181/33085328

[ref23] Drakhlis L. Biswanath S. Farr C.-M. Lupanow V. Teske J. Ritzenhoff K.. (2021). Human heart-forming organoids recapitulate early heart and foregut development. Nat. Biotechnol. 39, 737–746. doi: 10.1038/s41587-021-00815-9 33558697 PMC8192303

[ref25] Duval K. Grover H. Han L.-H. Mou Y. Pegoraro A. F. Fredberg J.. (2017). Modeling physiological events in 2D vs. 3D cell culture. Physiology 32, 266–277. doi: 10.1152/physiol.00036.2016, PMID: 28615311 PMC5545611

[ref26] Eichmüller O. L. Corsini N. S. Vértesy Á. Morassut I. Scholl T. Gruber V.-E.. (2022). Amplification of human interneuron progenitors promotes brain tumors and neurological defects. Science 375:eabf5546. doi: 10.1126/science.abf5546, PMID: 35084981 PMC7613689

[ref27] Elia A. Fossati S. (2023). Autonomic nervous system and cardiac neuro-signaling pathway modulation in cardiovascular disorders and Alzheimer’s disease. Front. Physiol. 14:1060666. doi: 10.3389/fphys.2023.1060666, PMID: 36798942 PMC9926972

[ref30] Fligor C. M. Lavekar S. S. Harkin J. Shields P. K. VanderWall K. B. Huang K.-C.. (2021). Extension of retinofugal projections in an assembled model of human pluripotent stem cell-derived organoids. Stem Cell Reports 16, 2228–2241. doi: 10.1016/j.stemcr.2021.05.009, PMID: 34115986 PMC8452489

[ref31] Frasier C. R. Zhang H. Offord J. Dang L. T. Auerbach D. S. Shi H.. (2018). Channelopathy as a SUDEP biomarker in Dravet syndrome patient-derived cardiac myocytes. Stem Cell Reports 11, 626–634. doi: 10.1016/j.stemcr.2018.07.012, PMID: 30146492 PMC6135724

[ref32] Fukuda K. Kanazawa H. Aizawa Y. Ardell J. L. Shivkumar K. (2015). Cardiac innervation and sudden cardiac death. Circ. Res. 116, 2005–2019. doi: 10.1161/CIRCRESAHA.116.304679, PMID: 26044253 PMC4465108

[ref34] Gordan R. Gwathmey J. K. Xie L.-H. (2015). Autonomic and endocrine control of cardiovascular function. World J. Cardiol. 7, 204–214. doi: 10.4330/wjc.v7.i4.204, PMID: 25914789 PMC4404375

[ref35] Habecker B. A. Anderson M. E. Birren S. J. Fukuda K. Herring N. Hoover D. B.. (2016). Molecular and cellular neurocardiology: development, and cellular and molecular adaptations to heart disease. J. Physiol. 594, 3853–3875. doi: 10.1113/JP271840, PMID: 27060296 PMC4945713

[ref36] Haisler W. L. Timm D. M. Gage J. A. Tseng H. Killian T. C. Souza G. R. (2013). Three-dimensional cell culturing by magnetic levitation. Nat. Protoc. 8, 1940–1949. doi: 10.1038/nprot.2013.125 24030442

[ref37] Hasan W. (2013). Autonomic cardiac innervation: development and adult plasticity. Organ 9, 176–193. doi: 10.4161/org.24892, PMID: 23872607 PMC3896589

[ref38] Henske E. P. Jóźwiak S. Kingswood J. C. Sampson J. R. Thiele E. A. (2016). Tuberous sclerosis complex. Nat. Rev. Dis. Prim. 2:16035. doi: 10.1038/nrdp.2016.35 27226234

[ref39] Hernandez I. Ramirez S. P. Salazar W. V. Mendivil S. Guevara A. Patel A.. (2023). A semi-three-dimensional bioprinted Neurocardiac system for tissue engineering of a cardiac autonomic nervous system model. Bioengineering 10:834. doi: 10.3390/bioengineering10070834, PMID: 37508861 PMC10376081

[ref40] Higurashi N. Uchida T. Lossin C. Misumi Y. Okada Y. Akamatsu W.. (2013). A human Dravet syndrome model from patient induced pluripotent stem cells. Mol. Brain 6:19. doi: 10.1186/1756-6606-6-19, PMID: 23639079 PMC3655893

[ref41] Hinton R. B. Prakash A. Romp R. L. Krueger D. A. Knilans T. K. (2014). Cardiovascular manifestations of tuberous sclerosis complex and summary of the revised diagnostic criteria and surveillance and management recommendations from the international tuberous sclerosis consensus group. J. Am. Heart Assoc. 3:e001493. doi: 10.1161/JAHA.114.001493, PMID: 25424575 PMC4338742

[ref42] Hofbauer P. Jahnel S. M. Papai N. Giesshammer M. Deyett A. Schmidt C.. (2021). Cardioids reveal self-organizing principles of human cardiogenesis. Cell 184, 3299–3317.e22. doi: 10.1016/j.cell.2021.04.034, PMID: 34019794

[ref43] Horsager J. Andersen K. B. Knudsen K. Skjærbæk C. Fedorova T. D. Okkels N.. (2020). Brain-first versus body-first Parkinson’s disease: a multimodal imaging case-control study. Brain 143, 3077–3088. doi: 10.1093/brain/awaa238, PMID: 32830221

[ref45] Imamura Y. Mukohara T. Shimono Y. Funakoshi Y. Chayahara N. Toyoda M.. (2015). Comparison of 2D- and 3D-culture models as drug-testing platforms in breast cancer. Oncol. Rep. 33, 1837–1843. doi: 10.3892/or.2015.3767, PMID: 25634491

[ref46] Kanazawa H. Fukuda K. (2022). The plasticity of cardiac sympathetic nerves and its clinical implication in cardiovascular disease. Front. Synaptic Neurosci. 14:960606. doi: 10.3389/fnsyn.2022.960606, PMID: 36160916 PMC9500163

[ref47] Kawano H. Okada R. Yano K. (2003). Histological study on the distribution of autonomic nerves in the human heart. Heart Vessel. 18, 32–39. doi: 10.1007/s003800300005, PMID: 12644879

[ref48] Kearney J. (2013). Sudden unexpected death in Dravet syndrome: SUDEP in Dravet mouse model. Epilepsy Currents 13, 264–265. doi: 10.5698/1535-7597-13.6.264, PMID: 24348122 PMC3854739

[ref49] Kim M.-H. Kim D. Sung J. H. (2021). A gut-brain Axis-on-a-Chip for studying transport across epithelial and endothelial barriers. J. Ind. Eng. Chem. 101, 126–134. doi: 10.1016/j.jiec.2021.06.021

[ref50] Langhans S. A. (2018). Three-dimensional in vitro cell culture models in drug discovery and drug repositioning. Front. Pharmacol. 9:6. doi: 10.3389/fphar.2018.00006, PMID: 29410625 PMC5787088

[ref51] Larsen H. E. Lefkimmiatis K. Paterson D. J. (2016). Sympathetic neurons are a powerful driver of myocyte function in cardiovascular disease. Sci. Rep. 6:38898. doi: 10.1038/srep38898, PMID: 27966588 PMC5155272

[ref52] Lewis-Israeli Y. R. Wasserman A. H. Gabalski M. A. Volmert B. D. Ming Y. Ball K. A.. (2021). Self-assembling human heart organoids for the modeling of cardiac development and congenital heart disease. Nat. Commun. 12:5142. doi: 10.1038/s41467-021-25329-5, PMID: 34446706 PMC8390749

[ref53] Lian X. T. Qing X. Wang Y. L. LeYuan G. Qian Y. Shao W. H.. (2023). Noradrenergic pathway from the locus Coeruleus to heart is implicated in modulating SUDEP. iScience 26:106284. doi: 10.1016/j.isci.2023.106284, PMID: 36968083 PMC10034435

[ref54] Limperopoulos C. Tworetzky W. McElhinney D. B. Newburger J. W. Brown D. W. Robertson R. L.. (2010). Brain volume and metabolism in fetuses with congenital heart disease: evaluation with quantitative magnetic resonance imaging and spectroscopy. Circulation 121, 26–33. doi: 10.1161/CIRCULATIONAHA.109.865568, PMID: 20026783 PMC2819908

[ref55] Lin S. Lin Y. Nery J. R. Urich M. A. Breschi A. Davis C. A.. (2014). Comparison of the transcriptional landscapes between human and mouse tissues. Proc. Natl. Acad. Sci. 111, 17224–17229. doi: 10.1073/pnas.1413624111, PMID: 25413365 PMC4260565

[ref56] Liu S. Xiang K. Yuan F. Xiang M. (2023). Generation of self-organized autonomic ganglion organoids from fibroblasts. IScience 26:106241. doi: 10.1016/j.isci.2023.106241, PMID: 36922996 PMC10009094

[ref57] Maden C. H. Gomes J. Schwarz Q. Davidson K. Tinker A. Ruhrberg C. (2012). NRP1 and NRP2 cooperate to regulate gangliogenesis, axon guidance and target innervation in the sympathetic nervous system. Dev. Biol. 369, 277–285. doi: 10.1016/j.ydbio.2012.06.026, PMID: 22790009 PMC3430865

[ref58] Malhotra J. D. Chen C. Rivolta I. Abriel H. Malhotra R. Mattei L. N.. (2001). Characterization of Sodium Channel α- and β-subunits in rat and mouse cardiac myocytes. Circulation 103, 1303–1310. doi: 10.1161/01.CIR.103.9.1303, PMID: 11238277

[ref59] Manea M. Comsa M. Minca A. Dragos D. Popa C. (2015). Brain-heart axis—review article. J. Med. Life 8, 266–271, PMID: 26351525 PMC4556904

[ref60] Marini V. Marino F. Aliberti F. Giarratana N. Pozzo E. Duelen R.. (2022). Long-term culture of patient-derived cardiac organoids recapitulated Duchenne muscular dystrophy cardiomyopathy and disease progression. Front. Cell and Develop. Biol. 10:878311. doi: 10.3389/fcell.2022.878311, PMID: 36035984 PMC9403515

[ref61] McQuillen P. S. Miller S. P. (2010). Congenital heart disease and brain development. Ann. N. Y. Acad. Sci. 1184, 68–86. doi: 10.1111/j.1749-6632.2009.05116.x 20146691

[ref62] Meléndez G. C. Li J. Law B. A. Janicki J. S. Supowit S. C. Levick S. P. (2011). Substance P induces adverse myocardial remodelling via a mechanism involving cardiac mast cells. Cardiovasc. Res. 92, 420–429. doi: 10.1093/cvr/cvr244, PMID: 21908647 PMC3211974

[ref63] Menasché P. Vanneaux V. Hagège A. Bel A. Cholley B. Parouchev A.. (2018). Transplantation of human embryonic stem cell–derived cardiovascular progenitors for severe ischemic left ventricular dysfunction. J. Am. Coll. Cardiol. 71, 429–438. doi: 10.1016/j.jacc.2017.11.047, PMID: 29389360

[ref64] Miller S. L. Huppi P. S. Mallard C. (2016). The consequences of fetal growth restriction on brain structure and neurodevelopmental outcome. J. Physiol. 594, 807–823. doi: 10.1113/JP271402, PMID: 26607046 PMC4753264

[ref65] Mishra S. Reznikov V. Maltsev V. A. Undrovinas N. A. Sabbah H. N. Undrovinas A. (2015). Contribution of sodium channel neuronal isoform Na _v_ 1.1 to late sodium current in ventricular myocytes from failing hearts. J. Physiol. 593, 1409–1427. doi: 10.1113/jphysiol.2014.278259, PMID: 25772296 PMC4376421

[ref66] Miura Y. Li M.-Y. Birey F. Ikeda K. Revah O. Thete M. V.. (2020). Generation of human striatal organoids and cortico-striatal assembloids from human pluripotent stem cells. Nat. Biotechnol. 38, 1421–1430. doi: 10.1038/s41587-020-00763-w, PMID: 33273741 PMC9042317

[ref67] Muguruma K. Nishiyama A. Kawakami H. Hashimoto K. Sasai Y. (2015). Self-Organization of Polarized Cerebellar Tissue in 3D culture of human pluripotent stem cells. Cell Rep. 10, 537–550. doi: 10.1016/j.celrep.2014.12.051, PMID: 25640179

[ref68] Neubert D. S. G. Seiffert N. Tovote P. (2023). Cerebellar contribution to the regulation of defensive states. Front. Syst. Neurosci. 17:1160083. doi: 10.3389/fnsys.2023.1160083, PMID: 37064160 PMC10102664

[ref69] Oh Y. Cho G.-S. Li Z. Hong I. Zhu R. Kim M.-J.. (2016). Functional coupling with cardiac muscle promotes maturation of hPSC-derived sympathetic neurons. Cell Stem Cell 19, 95–106. doi: 10.1016/j.stem.2016.05.002, PMID: 27320040 PMC4996639

[ref70] Olmsted Z. T. Paluh J. L. (2022). A combined human gastruloid model of cardiogenesis and neurogenesis. IScience 25:104486. doi: 10.1016/j.isci.2022.104486, PMID: 35721464 PMC9198845

[ref72] Pervolaraki E. Anderson R. A. Benson A. P. Hayes-Gill B. Holden A. V. Moore B. J. R.. (2013). Antenatal architecture and activity of the human heart. Interface Focus 3:20120065. doi: 10.1098/rsfs.2012.0065, PMID: 24427520 PMC3638472

[ref73] Pervolaraki E. Dachtler J. Anderson R. A. Holden A. V. (2018). The developmental transcriptome of the human heart. Sci. Rep. 8:15362. doi: 10.1038/s41598-018-33837-6, PMID: 30337648 PMC6194117

[ref74] Picollet-D’hahan N. Zuchowska A. Lemeunier I. le Gac S. (2021). Multiorgan-on-a-Chip: A systemic approach to model and decipher inter-organ communication. Trends Biotechnol. 39, 788–810. doi: 10.1016/j.tibtech.2020.11.014, PMID: 33541718

[ref76] Ravi M. Paramesh V. Kaviya S. R. Anuradha E. Solomon F. D. P. (2015). 3D cell culture systems: advantages and applications. J. Cell. Physiol. 230, 16–26. doi: 10.1002/jcp.24683, PMID: 24912145

[ref77] Reiner O. Sapir T. Parichha A. (2021). Using multi-organ culture systems to study Parkinson’s disease. Mol. Psychiatry 26, 725–735. doi: 10.1038/s41380-020-00936-8, PMID: 33154567 PMC7643717

[ref78] Rossi G. Broguiere N. Miyamoto M. Boni A. Guiet R. Girgin M.. (2021). Capturing Cardiogenesis in Gastruloids. Cell Stem Cell 28, 230–240.e6. doi: 10.1016/j.stem.2020.10.013, PMID: 33176168 PMC7867643

[ref79] Rossi A. Mikail N. Bengs S. Haider A. Treyer V. Buechel R. R.. (2022). Heart–brain interactions in cardiac and brain diseases: why sex matters. Eur. Heart J. 43, 3971–3980. doi: 10.1093/eurheartj/ehac061, PMID: 35194633 PMC9794190

[ref80] Schmidt C. Deyett A. Ilmer T. Haendeler S. Torres Caballero A. Novatchkova M.. (2023). Multi-chamber cardioids unravel human heart development and cardiac defects. Cell 186, 5587–5605.e27. doi: 10.1016/j.cell.2023.10.030, PMID: 38029745

[ref81] Sha L. Li Y. Zhang Y. Tang Y. Li B. Chen Y.. (2023). Heart-brain axis: association of congenital heart abnormality and brain diseases. Front. Cardiovascular Med. 10:1071820. doi: 10.3389/fcvm.2023.1071820, PMID: 37063948 PMC10090520

[ref82] Sheng J. A. Bales N. J. Myers S. A. Bautista A. I. Roueinfar M. Hale T. M.. (2021). The hypothalamic-pituitary-adrenal Axis: development, programming actions of hormones, and maternal-fetal interactions. Front. Behav. Neurosci. 14:601939. doi: 10.3389/fnbeh.2020.601939, PMID: 33519393 PMC7838595

[ref83] Siddiqui S. Wilpers A. Myers M. Nugent J. D. Fifer W. P. Williams I. A. (2015). Autonomic regulation in fetuses with congenital heart disease. Early Hum. Dev. 91, 195–198. doi: 10.1016/j.earlhumdev.2014.12.016, PMID: 25662702 PMC4821472

[ref84] Silva A. C. Matthys O. B. Joy D. A. Kauss M. A. Natarajan V. Lai M. H.. (2021). Co-emergence of cardiac and gut tissues promotes cardiomyocyte maturation within human iPSC-derived organoids. Cell Stem Cell 28, 2137–2152.e6. doi: 10.1016/j.stem.2021.11.007, PMID: 34861147

[ref85] Silvani A. Calandra-Buonaura G. Dampney R. A. L. Cortelli P. (2016). Brain–heart interactions: physiology and clinical implications. Philos. Trans. R. Soc. A Math. Phys. Eng. Sci. 374:20150181. doi: 10.1098/rsta.2015.0181, PMID: 27044998

[ref86] Simeone A. Avantaggiato V. Moroni M. C. Mavilio F. Arra C. Cotelli F.. (1995). Retinoic acid induces stage-specific antero-posterior transformation of rostral central nervous system. Mech. Dev. 51, 83–98. doi: 10.1016/0925-4773(95)96241-M, PMID: 7669695

[ref87] Skardal A. Aleman J. Forsythe S. Rajan S. Murphy S. Devarasetty M.. (2020). Drug compound screening in single and integrated multi-organoid body-on-a-chip systems. Biofabrication 12:025017. doi: 10.1088/1758-5090/ab6d36, PMID: 32101533

[ref88] Smits L. M. Reinhardt L. Reinhardt P. Glatza M. Monzel A. S. Stanslowsky N.. (2019). Modeling Parkinson’s disease in midbrain-like organoids. Npj Parkinson’s Disease 5:5. doi: 10.1038/s41531-019-0078-4, PMID: 30963107 PMC6450999

[ref89] Soares C. P. Midlej V. Oliveira M. E. W. D. Benchimol M. Costa M. L. Mermelstein C. (2012). 2D and 3D-organized cardiac cells shows differences in cellular morphology, adhesion junctions, presence of myofibrils and protein expression. PLoS One 7:e38147. doi: 10.1371/journal.pone.0038147, PMID: 22662278 PMC3360656

[ref90] Stavrakis S. University of Oklahoma Health Sciences Center, Oklahoma City, Oklahoma, USA, Po, S., & University of Oklahoma Health Sciences Center, Oklahoma City, Oklahoma, USA (2017). Ganglionated Plexi ablation: physiology and clinical applications. Arrhythmia Electrophysiol. Rev. 6, 186–190. doi: 10.15420/aer2017.26.1, PMID: 29326833 PMC5739885

[ref91] Tada M. Onodera O. Tada M. Ozawa T. Piao Y.-S. Kakita A.. (2007). Early development of autonomic dysfunction may predict poor prognosis in patients with multiple system atrophy. Arch. Neurol. 64, 256–260. doi: 10.1001/archneur.64.2.256, PMID: 17296842

[ref92] Tahsili-Fahadan P. Geocadin R. G. (2017). Heart–brain Axis: effects of neurologic injury on cardiovascular function. Circ. Res. 120, 559–572. doi: 10.1161/CIRCRESAHA.116.308446 28154104

[ref93] Takayama Y. Kushige H. Akagi Y. Suzuki Y. Kumagai Y. Kida Y. S. (2020). Selective induction of human autonomic neurons enables precise control of cardiomyocyte beating. Sci. Rep. 10:9464. doi: 10.1038/s41598-020-66303-3, PMID: 32528170 PMC7289887

[ref94] Tillo M. Ruhrberg C. Mackenzie F. (2012). Emerging roles for semaphorins and VEGFs in synaptogenesis and synaptic plasticity. Cell Adhes. Migr. 6, 541–546. doi: 10.4161/cam.22408, PMID: 23076132 PMC3547901

[ref95] Trapecar M. Wogram E. Svoboda D. Communal C. Omer A. Lungjangwa T.. (2021). Human physiomimetic model integrating microphysiological systems of the gut, liver, and brain for studies of neurodegenerative diseases. Sci. Adv. 7:eabd1707. doi: 10.1126/sciadv.abd1707, PMID: 33514545 PMC7846169

[ref96] Van Den Berge N. Ulusoy A. (2022). Animal models of brain-first and body-first Parkinson’s disease. Neurobiol. Dis. 163:105599. doi: 10.1016/j.nbd.2021.105599, PMID: 34952161

[ref97] Van Norman G. A. (2019). Phase II trials in drug development and adaptive trial design. JACC: Basic to Translational Sci. 4, 428–437. doi: 10.1016/j.jacbts.2019.02.005, PMID: 31312766 PMC6609997

[ref98] Varga Z. V. Ferdinandy P. Liaudet L. Pacher P. (2015). Drug-induced mitochondrial dysfunction and cardiotoxicity. Am. J. Phys. Heart Circ. Phys. 309, H1453–H1467. doi: 10.1152/ajpheart.00554.2015, PMID: 26386112 PMC4666974

[ref99] Voges H. K. Mills R. J. Elliott D. A. Parton R. G. Porrello E. R. Hudson J. E. (2017). Development of a human cardiac organoid injury model reveals innate regenerative potential. Development 144, 1118–1127. doi: 10.1242/dev.143966, PMID: 28174241

[ref100] Volmert B. Kiselev A. Juhong A. Wang F. Riggs A. Kostina A.. (2023). A patterned human primitive heart organoid model generated by pluripotent stem cell self-organization. Nat. Commun. 14:8245. doi: 10.1038/s41467-023-43999-1, PMID: 38086920 PMC10716495

[ref101] Will J. Siedentopf N. Schmid O. Gruber T. Henrich W. Hertzberg C.. (2023). Successful prenatal treatment of cardiac Rhabdomyoma in a fetus with tuberous sclerosis. Pediatric Reports 15, 245–253. doi: 10.3390/pediatric15010020, PMID: 36976727 PMC10059978

[ref102] Winbo A. Ramanan S. Eugster E. Jovinge S. Skinner J. R. Montgomery J. M. (2020). Functional coculture of sympathetic neurons and cardiomyocytes derived from human-induced pluripotent stem cells. Am. J. Phys. Heart Circ. Phys. 319, H927–H937. doi: 10.1152/ajpheart.00546.2020, PMID: 32822546

[ref103] Wood S. K. Valentino R. J. (2017). The brain norepinephrine system, stress and cardiovascular vulnerability. Neurosci. Biobehav. Rev. 74, 393–400. doi: 10.1016/j.neubiorev.2016.04.018, PMID: 27131968 PMC5083238

[ref104] Xiang Y. Tanaka Y. Cakir B. Patterson B. Kim K.-Y. Sun P.. (2019). HESC-derived thalamic organoids form reciprocal projections when fused with cortical organoids. Cell Stem Cell 24, 487–497.e7. doi: 10.1016/j.stem.2018.12.015, PMID: 30799279 PMC6853597

[ref105] Xiang Y. Tanaka Y. Patterson B. Kang Y.-J. Govindaiah G. Roselaar N.. (2017). Fusion of regionally specified hPSC-derived organoids models human brain development and interneuron migration. Cell Stem Cell 21, 383–398.e7. doi: 10.1016/j.stem.2017.07.007, PMID: 28757360 PMC5720381

[ref106] Yang D. Liu H.-Q. Liu F.-Y. Tang N. Guo Z. Ma S.-Q.. (2020). The roles of noncardiomyocytes in cardiac remodeling. Int. J. Biol. Sci. 16, 2414–2429. doi: 10.7150/ijbs.47180, PMID: 32760209 PMC7378633

[ref108] Yu W. McDonnell K. Taketo M. M. Bai C. B. (2008). Wnt signaling determines ventral spinal cord cell fates in a time-dependent manner. Development 135, 3687–3696. doi: 10.1242/dev.021899, PMID: 18927156

[ref109] Zambón D. Quintana M. Mata P. Alonso R. Benavent J. Cruz-Sánchez F.. (2010). Higher incidence of mild cognitive impairment in familial hypercholesterolemia. Am. J. Med. 123, 267–274. doi: 10.1016/j.amjmed.2009.08.015, PMID: 20193836 PMC2844655

[ref110] Zhang D. Lindsey S. E. (2023). Recasting current knowledge of human fetal circulation: the importance of computational models. J. Cardiovascular Develop. Dis. 10:240. doi: 10.3390/jcdd10060240, PMID: 37367405 PMC10299027

